# Design of a Serious Game for Handling Obstetrical Emergencies

**DOI:** 10.2196/games.5526

**Published:** 2016-12-21

**Authors:** Estelle Jean dit Gautier, Virginie Bot-Robin, Aurélien Libessart, Guillaume Doucède, Michel Cosson, Chrystèle Rubod

**Affiliations:** ^1^ Department of Gynecology Surgery Hopital Jeanne de Flandre University of Lille Lille cedex France; ^2^ Department of Gynecology Centre hospitalier de Seclin Seclin France; ^3^ 3DDUO Tourcoing France

**Keywords:** serious game, obstetric emergencies, gynecology

## Abstract

**Background:**

The emergence of new technologies in the obstetrical field should lead to the development of learning applications, specifically for obstetrical emergencies. Many childbirth simulations have been recently developed. However, to date none of them have been integrated into a serious game.

**Objective:**

Our objective was to design a new type of immersive serious game, using virtual glasses to facilitate the learning of pregnancy and childbirth pathologies. We have elaborated a new game engine, placing the student in some maternity emergency situations and delivery room simulations.

**Methods:**

A gynecologist initially wrote a scenario based on a real clinical situation. He also designed, along with an educational engineer, a tree diagram, which served as a guide for dialogues and actions. A game engine, especially developed for this case, enabled us to connect actions to the graphic universe (fully 3D modeled and based on photographic references). We used the Oculus Rift in order to immerse the player in virtual reality. Each action in the game was linked to a certain number of score points, which could either be positive or negative.

**Results:**

Different pathological pregnancy situations have been targeted and are as follows: care of spontaneous miscarriage, threat of preterm birth, forceps operative delivery for fetal abnormal heart rate, and reduction of a shoulder dystocia. The first phase immerses the learner into an action scene, as a doctor. The second phase ask the student to make a diagnosis. Once the diagnosis is made, different treatments are suggested.

**Conclusions:**

Our serious game offers a new perspective for obstetrical emergency management trainings and provides students with active learning by immersing them into an environment, which recreates all or part of the real obstetrical world of emergency. It is consistent with the latest recommendations, which clarify the importance of simulation in teaching and in ongoing professional development.

## Introduction

In most cases, pregnancy and childbirth are conducted without complications. However, when pathology occurs, sometimes severe and urgent, a fast and efficient care led by a perfectly trained team is needed. In these cases, the training is mostly performed on real patients under the supervision of a senior. However, emergency situation does not facilitate the learning process.

The occurrence of certain situations is therefore random and depends on the maternity services with which the student is affiliated.

The emergence of new technologies in the obstetrical field should lead to the development of learning applications, specifically for obstetrical emergencies. Indeed, according to the latest 2014 Haute Autorité de Santé recommendations, related to birth care quality and safety, simulation exercises are integrated into the team training for obstetrical emergencies [1]. Many trainings using simulation were designed to teach medical students and to develop their capacity to perform surgeries in the future. Effectiveness of this form of learning is now well established [2,3]. Furthermore, using virtual glasses-headphones coupling allows total immersion of the player in a 3D environment and affects both hearing and sight. This immersion is more complete as compared with a situation in which the player is just in front of a computer screen with keyboard and mouse control devices [4].

In the obstetrical field, many childbirth simulations have been recently designed for educational purposes [5]. However, to date, none of them have been integrated into a serious game, allowing a global “virtual patient” approach.

Our goal is to develop a new type of immersive, virtual reality serious game using virtual glasses. We describe the design of a new game engine, placing the student in some maternity emergency situations and delivery room simulations. In-game decision-making should lead, in the second step, to an accurate obstetrical gesture realization on a physical manikin.

## Methods

For several years, our obstetricians’ team has been working in partnership with an educational engineer.

Different pathological situations during pregnancy that have been targeted are as follows: threat of early spontaneous miscarriage, threat of preterm birth, forceps operative delivery for abnormal heart rate, and reduction of a shoulder dystocia.

This serious game targets different categories of learners.

Indeed, “spontaneous miscarriage” and “possible preterm birth” scenarios deal with 2 topics that are part of the French educational program of the medicine study second cycle [6]. They also form a part of the midwife educational program. Thus, these scenarios target a wide audience, composed of medicine students, obstetrical and gynecological internes and residents, and apprentice midwives. The “forceps operative delivery for abnormal fetal heart rate” scenario will permit the gynecology and obstetrics residents training.

Obstetricians and midwives must know the “shoulder dystocia” diagnosis and the related reduction gestures. The last 2 scenarios could be integrated as part of an ongoing medical training.

An obstetrician initially wrote a scenario based on a real clinical situation. All scenarios were designed following the same method. The first phase put the learner in an action scene, as a doctor. The second phase lead to making the diagnosis of the pathology. An actions diagram tree referring to all the possible actions enables the students to progress following a clinical reasoning. Players can have access to various additional clinical elements, for example, the possibility to perform an ultrasound, a biological assessment, consult medical records, or fetal monitoring. Once the diagnosis is made, different treatments are offered to learners.

We want to reach the most real-like situation by offering students the maximum dialog choices and additional tests.

In order to offer a certain freedom in action, a complex actions diagram tree lead in-game actions and allows students to make their own choices in the game.

**Figure 1:**
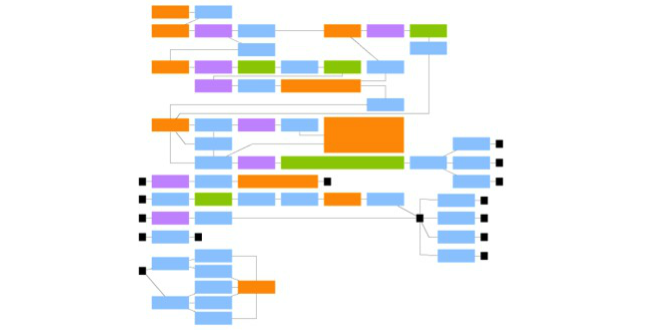
Orange: environment changes and specific content display; blue: player decision making; purple: interaction with object; green: game automatic feedback; black: checkpoint.

Both an educational engineer and a doctor have designed this actions tree. It contains all the possible actions, which are given to players in the game. Thanks to this method, the scenario moves away from a linear path, and allows students to learn through attempts and errors. All these actions diagram trees are designed with a diagramming software, such as Ed Graph Editor, (see Figure 1).

This actions diagram has been evaluated for its relevance by doctors with different knowledge levels. Thus, medicine students, obstetrical residents, and hospital practitioners tested it. It was aimed to highlight possible misunderstandings or inconsistencies, which may persist in the scenario realization. This will also enable us to expand the range of options offered to players. Some answers may not have been initially envisaged and might be relevant in diagnostic or therapeutic procedures.

**Figure 2:**
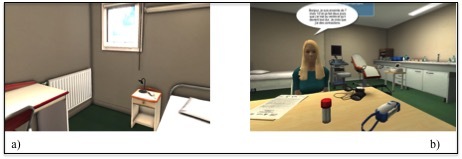
Elaboration of a graphic environment: (a) 3D modeled graphic universe, based on hospital photographic references: guard room and (b) obstetrical emergencies unit.

The graphic universe has been fully 3D modeled and is based on hospital photographic references. Different views have been taken in some strategic locations such as obstetrical emergencies unit, guardrooms, and delivery rooms (see Figure 2). So far, 2 locations have been 3D modeled in order to compose the environment of the 4 scenarios. “Spontaneous miscarriage” and “possible preterm birth” scenarios take place in the obstetrical emergencies unit. “Forceps operative delivery for fetal abnormal heart rate” and “reduction of a shoulder dystocia” scenarios take place in a delivery room. These different locations are 3D modeled using “Autodesk 3ds Max” software.

Game development was carried out following 2 distinct phases.

First, it was necessary to develop a game engine. Indeed, once the actions tree design was completed, each actions branch had to be linked to in-game actions. Each action contains, via the game engine, the action description, the previous actions (leading to this action), and the following actions (unlocked by this action). Some other information can also be set up, such as corresponding interactive objects, code names, and so on. Second, indicated actions are connected with the graphic universe. For example, ultrasound actions type appeared by clicking on the ultrasound equipment, respecting the timeline and the causes or consequences relationship established in the actions tree diagram.

Players are immersed in virtual reality thanks to the Oculus Rift technology, a virtual reality device designed by the Oculus VR Company. The device looks like a mask covering eyes and can be strapped to the face at the rear of the head. A digital screen is placed a few centimeters in front of each eye, perpendicular to the sight line. This screen displays a stereoscopic picture, digitally distorted by 2 lenses located in front of each eye, in order to inverse the optical distortion. It expands the visual field and the definition in front of the fovea. The screen is placed in the focal plane of these lenses. The created virtual picture is projected to infinity. Various sensors detect user head movements, which make a real-time picture adaptation on the screen possible and produce a total immersion into the rendered scene.

A score, which can be either positive or negative, is set for every possible in-game action. A good action is positively rewarded, and conversely, bad actions are negatively rewarded. Therefore, certain choices would be rewarded, and others would be penalized. Score points setting depend on the student diagnostic process and its relevance. For example, if the student immediately led a biological assessment before interrogating or examining the patient, the student will be rewarded less score points than a student who follows a correct approach, with a medical logic (interrogation, followed by clinical tests, and additional tests). In delivery room scenarios, a concept of penalty depending on the student’s decision time is added.

**Figure 3:**
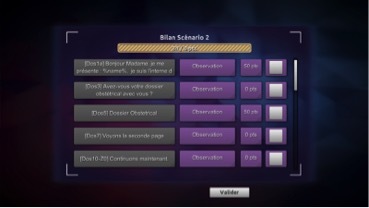
Score table: personal and downloadable score table of the player.

All these actions are then summarized at the end of the game in a score table (see Figure 3). The score table is divided into 5 sections. On the top, the global player score is provided that the player earned on the entire scenario. The first column reminds each action he or she performed, the second shows the actions category, the third shows the score associated with this particular action, and the last column often leads to additional content (videos, articles, and courses).

Every time a session ends, a personal and downloadable assessment of the player is edited through a spreadsheet software (Excel-like). Thus, the player can see his mistakes and successes at the end of the game. A scoring system permits the player assessment. The learning assessment is a two-step process. The first step is made through the game based on the choices the player virtually made and for which he or she has been given points (positive and negative) assigning the player a final grade. The second step occurs through the actual debrief at the end of the learning session, with the teacher who will go through the blunt assessment given by the game.

## Results

All the scenarios mentioned above have been developed.

Two scenarios take place in obstetrical emergencies unit. The 2 selected pathologies are frequent motives for consultations: spontaneous miscarriage and possible preterm birth [7].

The player is in an emergency room. He or she has access to all the necessary equipment in order to solve the clinical case: personal medical records, cardiotocography, lookup table, ultrasound device, tensiometer, thermometer, tubes for biological samples, or even urine sample bottles for urinalysis strips uses. Each element has a specific role in the scenario.

The threat of preterm birth scenario is a simple situation in which a patient comes to obstetrical emergencies unit for pelvic pain at 30 weeks of gestation. We have created a virtual dialogue between the patient and the learner or player in order to provide him with the necessary information on diagnosis. The learner can also consult the personal medical records in order to access various data such as patient history, allergies, and pregnancy monitoring. If the player wishes, he or she can perform an ultrasound, or consult the fetal heart rate and tocography. If so, he or she will have an access to ultrasound images we have included in the scenario (cervical length and estimated fetal weight). The purpose of these additional elements is to guide the learner toward a possible preterm birth diagnosis. However, only the player can choose if he or she realizes (or not) a medical interrogation and additional examinations. He or she can then choose among several therapeutic treatments. To some extent, the player can constantly access the ongoing diagnosis before choosing the appropriate treatment and modify it if he or she wants. However, there are points of no return: Once he or she reaches one, the player cannot go back would he or she want to obtain additional information and modify his diagnosis. The diagnostic process is estimated at 20 minutes, which is the time amount allotted to the player to solve the case. With this countdown system, we want to recreate the stressful conditions of emergency services (average consulting time).

The “spontaneous miscarriage scenario” takes place in the same environment and is based on the same game action engine, with a similar diagnostic approach. A patient, at the first trimester of pregnancy, comes for metrorrhagia. The player can realize detailed examinations, ultrasound, and biological check-up (pregnancy blood test, complete blood count). He or she should eventually make a spontaneous miscarriage diagnosis and choose the adapted therapy. Regarding the scenarios taking place in the delivery room, we needed to create a new graphic environment.

An obstetrical unit midwife calls the player and asks him to come into the delivery room. He or she can consult the personal medical records, the monitoring (showing an abnormal fetal heart rhythm and expulsive efforts), ultrasound, and has access to various obstetrical tools.

Regarding “forceps operative delivery through forceps for abnormal heart rate scenario,” the player can lead a patient and a midwife interrogation, perform a vaginal examination, an ultrasound, and collect some information about fetal head engagement and orientation. The goal is to achieve as soon as possible the abnormal fetal heart rate diagnosis requiring forceps operative delivery. The decision must be taken within 10 minutes in order to represent the urgency of this type of situation.

The “reduction of a shoulder dystocia scenario” takes place in the same way. The same additional examination possibilities are offered to the player. Most of them are useless, and even have a negative impact and result in a waste of time because the shoulder dystocia diagnosis is purely clinical. The player goal is to reach this diagnosis as soon as possible and choose among various proposed gestures.

## Discussion

### Principal Findings

We designed a new type of immersive serious game, using a new game engine. It supports the Oculus Rift technology, allows the integration of new scenarios with minimal effort, especially in the gynecologic field, and could be linked to the medical training manikin. It has been designed so that it is sensor-equipped to allow gesture realization.

The use of new simulation technologies has been widespread in the obstetrical field for the last ten years. However, to our knowledge, there is no publication about serious games used as pedagogic tools for learning in obstetrical emergency situations.

Nowadays, learning through simulation mostly concerns trainings related to breast and pelvic clinical examination as well as to postpartum hemorrhage management [8]. This learning form is commonly linked to the technical skills reinforcement, especially with postexercise debriefing. Through the game, our educational tool can help stimulate the motivation of medicine students, obstetrical interns, residents, and midwifery students by offering the possibility to face frequent emergency situations, which require quick management answers and are based on essential knowledge.

Some commercial or nonprofit platforms already exist but have not been totally adapted to the medical training needs. For instance, these game engines do not support the Oculus Rift technology. Based on our training needs, we created our own specific tool. It allows the incorporation of a complex diagram tree, based on a real clinical situation, which offers a multiple action combination and leads to a realistic and immersive game.

Moreover, the project must lead to the integration of a medical training manikin, be sensor-equipped, and link to the Serious Game, to allow obstetrical gesture training with real-time feedbacks. Our game engine should therefore be custom designed in regard with this evolution. Serious games developments in the medical field are often very specific to each project. Few of them use a game engine, which allows creating new scenarios with minimal efforts, especially in the gynecologic field. Our work led to the development of a new game engine, which allows integrating, quickly and easily, new scenarios, both in the gynecologic and obstetric fields, as well as in other medical fields, without requiring the intervention of a programmer. Each scenario can be integrated into a new graphic environment, corresponding to various hospital locations, using already created graphic assets. Moreover, few serious games offer a complete immersion in the medical field currently. Immersion, through the Oculus Rift, can increase the realism of a clinical situation and the involvement of the player [9].

Learning by playing seems to be a solid method to gain better appropriation for the learner [10]. Serious games, mostly developed for surgical skills, have been tested to prove their validity [11-13]. Serious games seem to better enable the learner to feel immersed, to improve their confidence, and to enhance their clinical skills [12]. Serious games offer an innovative approach and seem more attractive than the “old fashioned way” of learning. Nevertheless, in order to do so, it is important to cooperate in designing and validating a serious game for a specific educational problem [14,15]. That is why we combined both the work and the skills of a pedagogic engineer, a game designer, a medical doctor, and a medical professor.

Our educational method aims at learning various common gynecological pathologies through realistic virtual situations. The game provides the learner with the opportunity to think and to follow a patterned diagnosis approach. Thanks to the postgame score analysis, the learner can evaluate himself and get debriefed about the mistakes he or she committed during the game session.

Naturally, this tool has to be properly tested on a student’s sample in order to validate its performances and demonstrate its pedagogic potential, and also evaluate the good learning acquisition. Thanks to the game and to the learning session with the debrief-time.

Even today, *serious game* is not a well-defined concept. So far, it has many definitions, which differ from author to author [16-20]. Our work matched with the definition of Julian Alvarez that defines serious games as any “computer application whose initial intention is to combine, with consistency, both serious aspects with fun spring from video game” [18]. We have described a system that meets this definition by using scoring system, action and decision tree, stressful and immersive graphic environment narrative systems, and game mechanics based on flow and game design theory (especially from point and click adventure game type). This Serious Game is developed as a part of a continuing medical training, funded by Lille2 University. It is used within the framework of learning sessions, managed by a professor. A medical teacher carries out the briefing and the debriefing. In this context, the game is only a support, used to initiate a dialogue involving the player’s own knowledge. However, this game can be also used as a “standalone game.” The final report screen allows nonetheless a basic assessment, even without learning sessions. The scoring system, initially designed by a doctor, allows the player to visualize his mistakes, and to get the “correct answer” related to the clinical situation he or she just experienced. Prior to any training, we will use these sessions as an opportunity to make A-B testing (with and without the game) and evaluate its usability, through a form filled by the student at the end of each session.

So far, delivery room scenarios end at the obstetrical gesture choice.

Thereafter, we would allow the learner to realize these technical gestures on a sensor-equipped obstetrical anatomic manikin, which tracks each learner’s gestures.

We are working on a virtual simulator of the pregnant woman’s pelvic system in order to allow gestures on a digital model. The game action engine of our serious game can also integrate new future scenarios such as ectopic pregnancy, operative delivery for obstructed labor, and/or vacuum extraction.

### Conclusion

Simulation teaching offers active learning, conducted by the learner, immersed in an environment recreating all or part of the real world, promoting knowledge integration and technical and behavioral skills in a short time.

Our serious game is part of this type of educational training, and offers a new perspective for obstetrical emergency learning. We need to test it on a student sample to validate its pedagogic potential in order to justify its integration into an obstetrical learning program.

We want to develop it by creating some new scenario in the gynecologic and obstetrical fields to extend its pedagogical impact.
